# Optimized design of permanent magnet for disk permanent magnet governor

**DOI:** 10.1038/s41598-023-47047-2

**Published:** 2023-11-15

**Authors:** Manman Xu, Yili Fu, Long Shao, Xiangdong Wang, Yimin Lu, Chuqing Cao

**Affiliations:** 1 School of Mechanical Engineering, Anhui Polytechnic University, Wuhu, 241000 China; 2 Wuhu HIT Robot Technology Research Institute Co., Ltd, Wuhu, 241000 China; 3 Harbin Institute of Technology, Harbin, 150001 China; 4 Wuhu Magnetic Wheel Transmission Technology Co., Ltd, Wuhu, 241000 China

**Keywords:** Mechanical engineering, Applied physics

## Abstract

To research the magnetic field and mechanical characteristics of the permanent magnet governor, the static magnetic field of the sector permanent magnet is analyzed by the molecular current method in the permanent magnet governor. The magnetic flux distribution is acquired at any spatial position. Comparing the analytical value with the simulation value, the results show that they are basically consistent. Based on the analytical formula, the influence of the radial position, radial length, thickness, and pole number on the magnetic induction intensity of the permanent magnet governor is studied. Thus, it provides the theoretical reference for the structural optimized design. At the same time, a test bench was set up to measure the magnetic induction intensity. The calculation and experimental results show that the magnetic induction strength of the permanent magnet is increased by 27.5%, the axial component of the air gap flux density is increased by 14.3%, and the permanent magnet material is reduced by 7.84%.

## Introduction

Researchers have developed a variety of permanent magnet devices at home and abroad, the permanent magnet governor is one of them^1^. Permanent magnet governors can be categorized as disk governors and cylinder governors based on their structural types. Disk governors are the most popular in engineering because of their simple construction and easy installation. With high reliability, high efficiency and energy saving, vibration isolation, light load starting, and adaptable ability in harsh environments, permanent magnet governor have attracted more and more attention from energy-consuming enterprises as a national key energy-saving technology^2^. At present, the research of permanent magnet governors mainly focuses on performance research, structural design optimization, and energy-saving applications. By researching the transient and steady-state of axial field eddy currents, LUBIN T used the vector magnetic potential approach to obtain the analytical equation of torque, which is highly consistent with the finite element simulation results^3^; Liu et al.^4^ proposed a hybrid high-efficiency permanent magnet governor, and established the mathematical model of temperature field and conducted simulation analysis. The results showed that the casing temperature with cooling ribs was significantly lower than that without cooling ribs; Wang et al., used a bidirectional coupling method to simulate and analyze the temperature field. The results showed that the bidirectional coupling simulation was more accurate than the unidirectional coupling simulation^5^; Seo et al., established an analytical model for the synchronous permanent magnet coupler based on the vector magnetic potential method. The analytical model accuracy was confirmed by comparison with the FEM analysis and experimental results^6^; Wu et al.^7^ established a numerical model to study the structural parameters effect of the disk permanent magnet governor on the speed regulation performance, and the accuracy of the derived equation was proved by comparison with the FEM simulation results; Jin et al.^8^ obtained the influence relationship of an air gap, conductor shape, poles number and speed difference on the output torque of permanent magnet governor by using Ansoft Maxwell software; Li used the equivalent magnetic circuit method to study the magnetic field distribution, and the structural parameters influence on transmission performance of the axial radial combined permanent magnet governor. The results show that the effectiveness and model accuracy are in good agreement with the finite element method and experimental results^9^. Zhang and Li^10^ and Yao^11^ have studied the energy-saving principle, energy-saving benefits, and technical characteristics of permanent magnet regulators by the equivalent magnetic circuit method, which showed that it has obvious advantages compared with other speed regulation technologies. However, at present, most studies on characteristics and parameter optimization are based on finite element software or a large number of experiments and experiences. Basic research is relatively lacking, and analytical methods are rarely used for analysis, design, and optimization.

Currently, the vector magnetic potential method and the equivalent magnetic circuit method are the main numerical simulation methods in engineering. The vector magnetic potential method derives the differential equations about the magnetic vector potential from the Maxwell equations. Then, the finite element or boundary element method is used to solve the problem numerically. When calculating the external magnetic field distribution, it is necessary to know the permeability or magnetization curve of the permanent magnet in the magnetization direction. However, it is difficult to determine these parameters. The magnetic circuit method converts the magnetic field’s calculation into the magnetic circuit’s calculation by reducing the magnetic field to the magnetic circuit equivalent to a circuit in an electric field. The calculation process is simple, and the accuracy is not high. In contrast, the molecular current method concentrates the bound current on the surface of the permanent magnet. It simplifies the model to calculate the spatial distribution of the magnetic field by the equivalent current. This method has simple principles and high precision.

To solve the above problems, the molecular current method was used in this paper to analyze the magnetic induction intensity of the permanent magnet governor^12^. And the accuracy of the analytical solution was verified by comparing it with the finite element simulation results, and the distribution law of the magnetic field was analyzed. Based on the analytical formula, the influence of the change of permanent magnet parameters on the permanent magnet governor was analyzed, and the influence law of the parameters of the permanent magnet on the magnetic induction intensity was obtained.

## Structure and principle of permanent magnet governor

**Figure 1:**
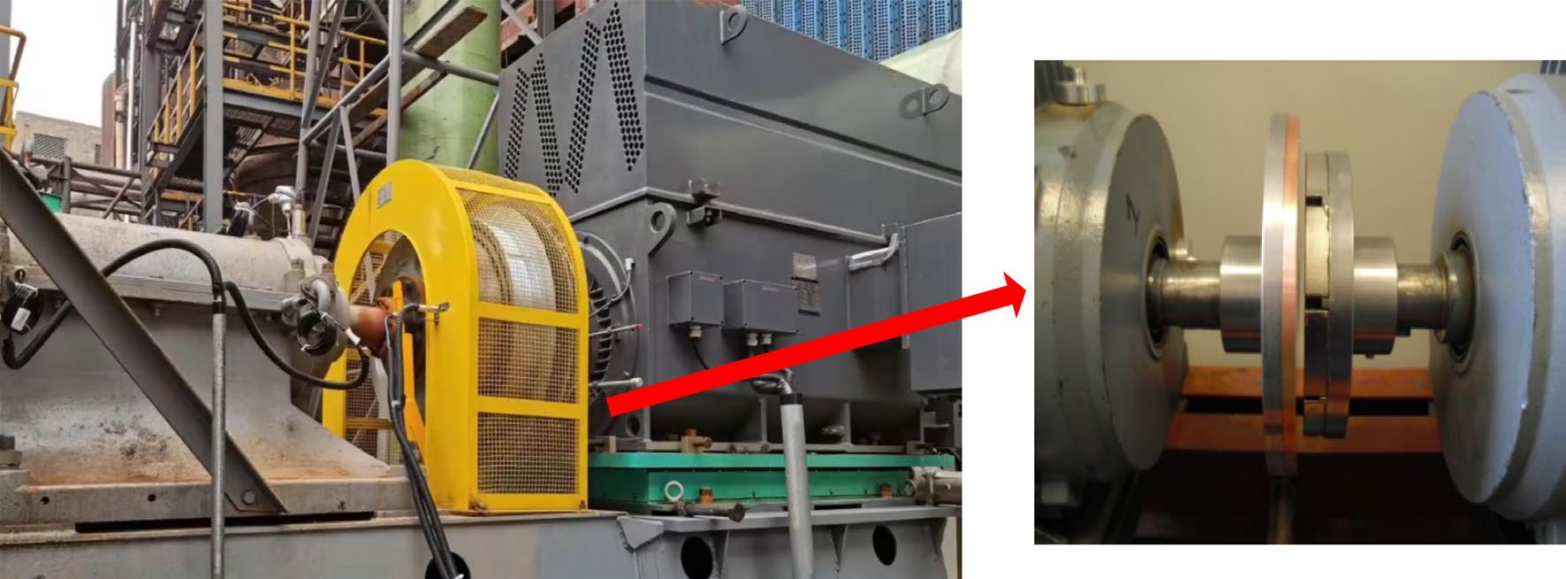
The physical picture of permanent magnet governor.

**Figure 2:**
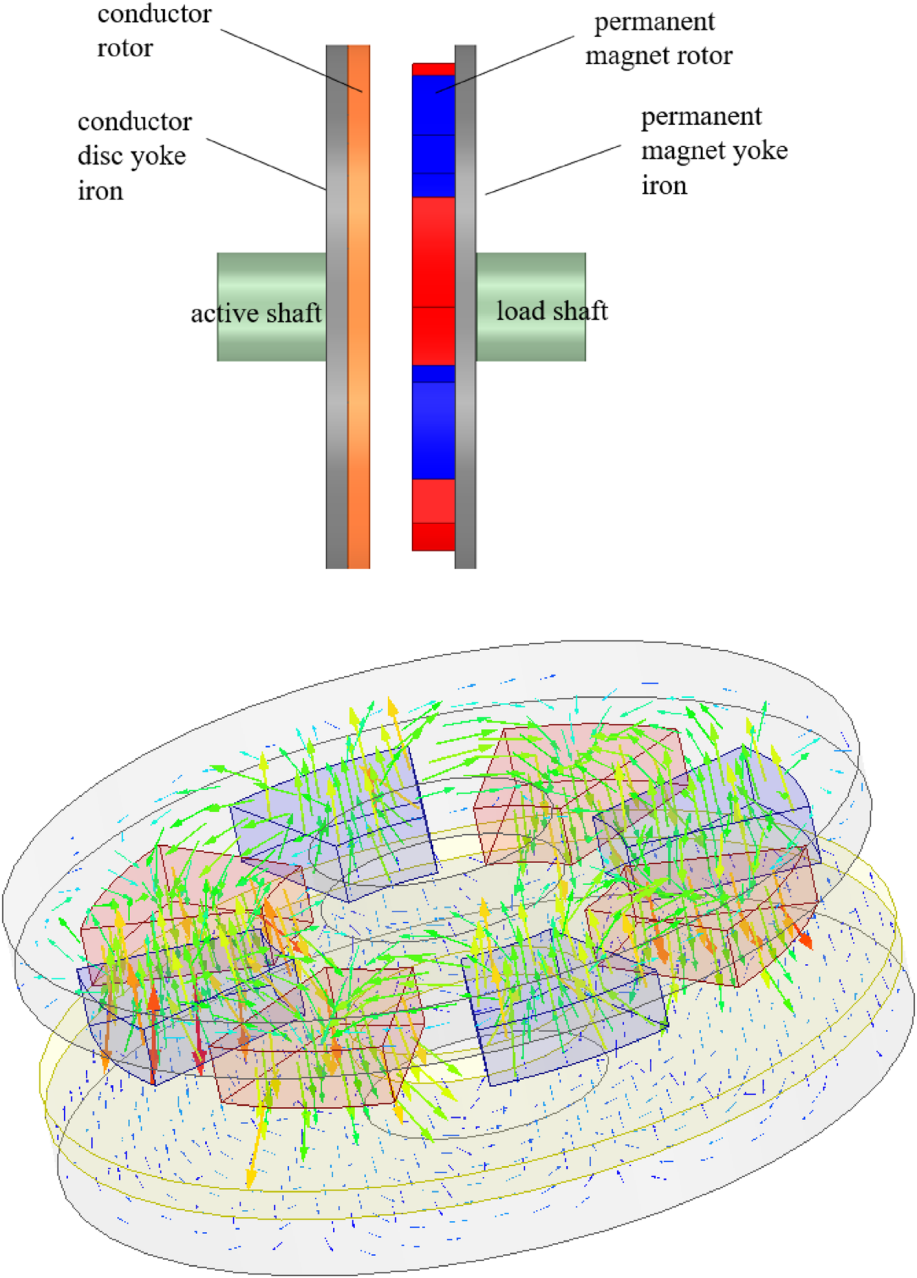
Principle model of permanent magnet governor.

The permanent magnet governor is mainly consisting of a permanent magnet rotor, conductor rotor, and regulating mechanism as shown in Fig. 1. It mainly includes the active shaft, load shaft, permanent magnet rotor, conductor rotor, yoke iron, and air gap adjustment mechanism. Its operation principle is that there is a speed difference between the permanent magnet and the conductor rotor. When the two parts move relative to each other, the conductor rotor cuts the magnetic induction lines to generate eddy currents. The induced magnetic field generated by the eddy currents and the magnetic field generated by the permanent magnets are coupled to each other to transmit torque and drive load as shown in Fig. 2^13^.

## Mathematical model based on molecular current method

### Theoretical analysis

**Figure 3:**
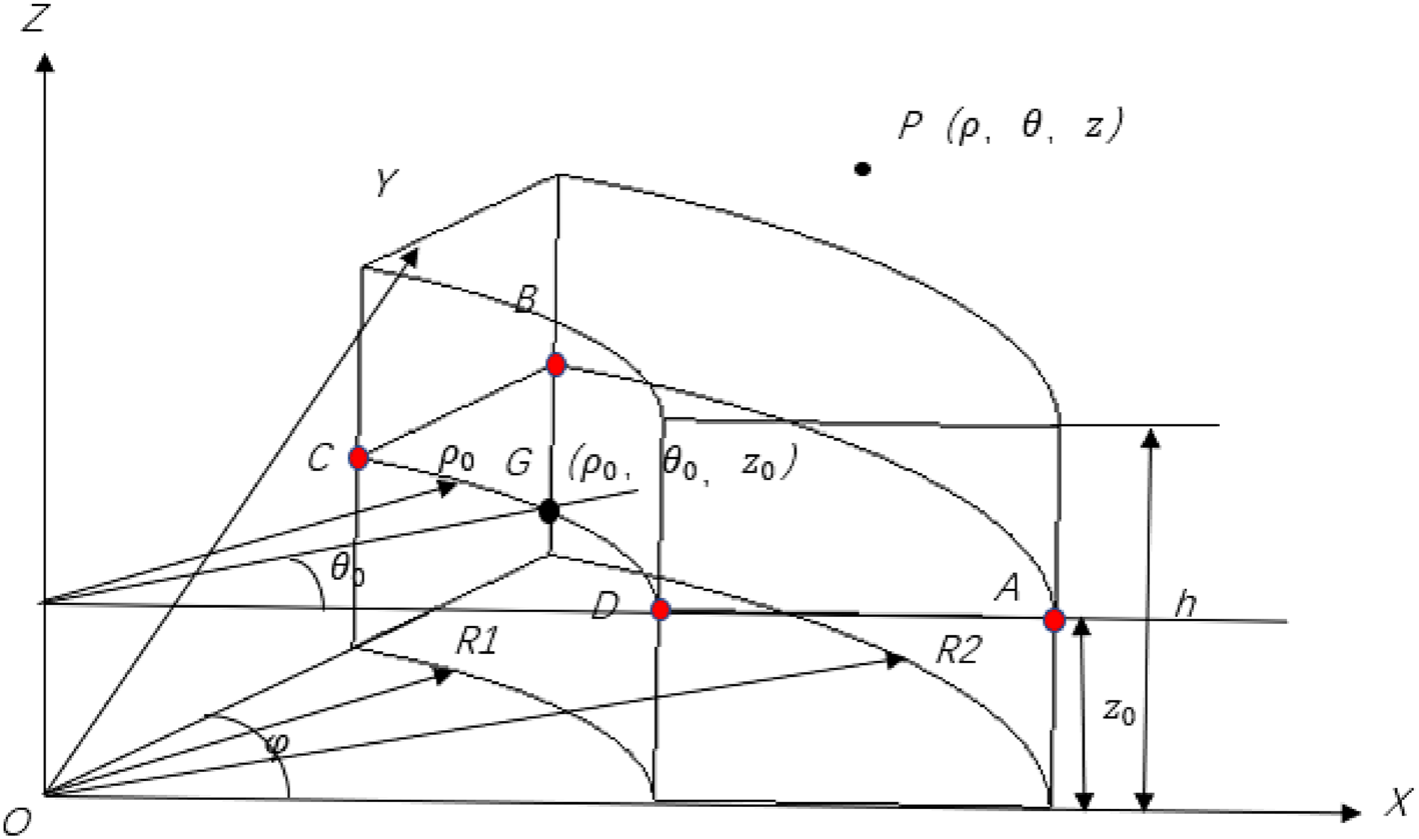
Equivalent current model.

Figure 3 shows the molecular circulating current model of permanent magnets. Assuming that the sector permanent magnet is fully and uniformly magnetized in the axial direction and reaches a saturation state. According to Ampere’s law of molecular circulation, macroscopically, the permanent magnet only has surface current but no body current. The magnetic induction intensity generated by the current loop is considered the superposition of the magnetic induction generated by each current element. The single-turn coil trajectory is continuous but not smooth and cannot be integrated continuously, so the equivalent current coil is divided into a superposition of four smooth current segments.

In Fig. 3, *R*_1_ and *R*_2_ are the inner and outer diameters of the permanent magnet, respectively; *h* is the height of the permanent magnet; *φ* is the angle of the permanent magnet; $P\left( {x,y,z} \right)$ or $\left( {\rho ,\theta ,z} \right)$ is any point in space, which is denoted as the field point $G\left( {x_{0} ,y_{0} ,z_{0} } \right)$ or $\left( {\rho_{0} ,\theta_{0} ,z_{0} } \right)$ is any point on the permanent magnet, which is denoted as the source point; The current element at the source point G can be determined by Biot Savart law and the current intensity ${\text{I}} = {\text{J}}dz_{0}$.

According to the calculation of the current element magnetic field provided by Biot Savart law, the magnetic induction of the current ring at any point P in space is following.

1 $$ {\varvec{B}} = \frac{{\mu_{0} }}{4\pi }\iiint {\frac{{Id{\varvec{l}} \times {\varvec{r}}}}{{\left| {\varvec{r}} \right|^{3} }}dV} $$

where, $\mu_{0}$ is the vacuum permeability, *dl* is the microlinear element, *l* is the integral path, $r$ is the vector diameter from the source to the field point P, and V is the distribution space volume.

The magnetic field B is generated by a permanent magnet at any point in space.

2 $$ {\varvec{B}} = B_{x} {\varvec{i}} + B_{y} {\varvec{j}} + B_{z} {\varvec{k}} = \mathop \smallint \nolimits_{0}^{{\varvec{h}}} {\varvec{dB}}_{{\varvec{x}}} {\varvec{i}} + {\varvec{dB}}_{{\varvec{y}}} {\varvec{j}} + {\varvec{dB}}_{{\varvec{z}}} {\varvec{k}} $$

where, *dB*_*x*_, *dB*_*y*_ and *dB*_*z*_ are the magnetic field components generated by the current element at point P in three directions, respectively.

3 $$ d{\varvec{B}} = \frac{{\mu_{0} }}{4\pi }\frac{{Jdz_{0} d{\varvec{l}} \times {\varvec{r}}}}{{\left| r \right|^{3} }} $$

4 $$ {\varvec{r}} = \left( {x - x_{0} } \right){\varvec{i}} + \left( {y - y_{0} } \right){\varvec{j}} + \left( {z - z_{0} } \right){\varvec{k}}{ = }\left( {\rho \cos \theta - \rho_{0} \cos \theta_{0} } \right){\varvec{i}} + \left( {\rho \sin \theta - \rho_{0} \sin \theta_{0} } \right){\varvec{j}} + \left( {z - z_{0} } \right){\varvec{k}} $$

5 $$ \left| {\varvec{r}} \right| = \sqrt {\left( {x - x_{0} } \right)^{2} + \left( {y - y_{0} } \right)^{2} + \left( {z - z_{0} } \right)^{2} } = \sqrt {\rho^{2} + \rho_{0}^{2} + 2\rho \rho_{0} \cos \left( {\theta - \theta_{0} } \right) + \left( {z - z_{0} } \right)^{2} } $$

For the AB segment,

6 $$ d{\varvec{l}} = - \sin \theta_{0} dl{\varvec{i}} + \cos \theta_{0} dl{\varvec{j}} = - \rho_{0} \sin \theta_{0} {\varvec{i}} + \rho_{0} \cos \theta_{0} {\varvec{j}} $$

There is a relationship of $\rho_{0} = R_{2}$ among r and $\left| {\varvec{r}} \right|$.

Substituting the above equations to obtain the magnetic induction produced by the current element at point *P*.

7 $$ d{\varvec{B}}_{{{\varvec{AB}}}} = \frac{{\mu_{0} Jdz_{0} }}{4\pi }\mathop \smallint \nolimits_{0}^{\varphi } \frac{{\left[ {R_{2} \left( {z - z_{0} } \right)\cos \theta_{0} {\varvec{i}} + R_{2} \left( {z - z_{0} } \right)\sin \theta_{0} {\varvec{j}} + \left( {R_{2}^{2} - \rho R_{2} \cos \left( {\theta - \theta_{0} } \right)} \right){\varvec{k}}} \right]d\theta_{0} }}{{\left[ {\rho^{2} + R_{2}^{2} + 2\rho R_{2} \cos \left( {\theta - \theta_{0} } \right) + \left( {z - z_{0} } \right)^{2} } \right]^{\frac{3}{2}} }} $$

For the BC segment,

8 $$ d{\varvec{l}} = \cos \theta_{0} dl{\varvec{i}} + \sin \theta_{0} dl{\varvec{j}} = \rho_{0} \sin \theta_{0} {\varvec{i}} + \rho_{0} \cos \theta_{0} {\varvec{j}} $$

There is a relationship of $\theta_{0} = \varphi$ among r and $\left| {\varvec{r}} \right|$.

Substituting the above equations to obtain the magnetic induction produced by the current element at point *P*.

9 $$ d{\varvec{B}}_{{{\varvec{BC}}}} = \frac{{\mu_{0} Jdz_{0} }}{4\pi }\mathop \smallint \nolimits_{R1}^{R2} \frac{{\left[ {\left( {z - z_{0} } \right)\sin \varphi {\varvec{i}} - \left( {z - z_{0} } \right)\cos \varphi {\varvec{i}} + \rho \sin \left( {\theta - \varphi } \right){\varvec{k}}} \right]d\rho_{0} }}{{\left[ {\rho^{2} + \rho_{0}^{2} + 2\rho \rho_{0} \cos \left( {\theta - \varphi } \right) + \left( {z - z_{0} } \right)^{2} } \right]^{\frac{3}{2}} }} $$

By analogy, the magnetic induction intensity of CD and DA segments can be obtained. According to the current direction superposition and integration of magnetization direction z. The total magnetic fields generated by permanent magnet ABCD at any point *P(x, y, z)* in its external space can be obtained. Among them,

10 $$ {\text{B}}_{{\varvec{z}}} = \frac{{\mu_{0} J}}{4\pi }\mathop \smallint \nolimits_{0}^{h} \left[ {{\text{A}}_{1} + {\text{A}}_{2} {\text{ + A}}_{3} {\text{ + A}}_{4} } \right]dz_{0} $$

11 $$ {\text{A}}_{1} = \mathop \smallint \nolimits_{0}^{\varphi } \frac{{\left[ {R_{1}^{2} - \rho R_{1} \cos \left( {\theta - \theta_{0} } \right)} \right]d\theta_{0} }}{{\left[ {\rho^{2} + R_{1}^{2} - 2\rho R_{1} \cos \left( {\theta - \theta_{0} } \right) + \left( {z - z_{0} } \right)^{2} } \right]^{\frac{3}{2}} }} $$

12 $$ {\text{A}}_{{2}} = \mathop \smallint \nolimits_{R1}^{R2} \frac{{\rho \sin \left( {\theta - \varphi } \right)d\rho_{0} }}{{\left[ {\rho^{2} + \rho_{0}^{2} - 2\rho \rho_{0} \cos \left( {\theta - \varphi } \right) + \left( {z - z_{0} } \right)^{2} } \right]^{\frac{3}{2}} }} $$

13 $$ {\text{A}}_{3} = \mathop \smallint \nolimits_{0}^{\varphi } \frac{{\left[ {R_{2}^{2} - \rho R_{2} \cos \left( {\theta - \theta_{0} } \right)} \right]d\theta_{0} }}{{\left[ {\rho^{2} + R_{2}^{2} - 2\rho R_{2} \cos \left( {\theta - \theta_{0} } \right) + \left( {z - z_{0} } \right)^{2} } \right]^{\frac{3}{2}} }} $$

14 $$ {\text{A}}_{4} = \mathop \smallint \nolimits_{R1}^{R2} \frac{{\rho \sin \theta d\rho_{0} }}{{\left[ {\rho^{2} + \rho_{0}^{2} - 2\rho \rho_{0} \cos \theta + \left( {z - z_{0} } \right)^{2} } \right]^{\frac{3}{2}} }} $$

$$ J_{s} = {\varvec{M}} \times {\mathbf{n}} $$

where, M is the magnetization, n is the unit vector of the outer normal on the side surface of the magnet.

From the above equations, it can be seen that the magnetic induction intensity distribution of the sector permanent magnet is related to the permanent magnet parameters, spatial location, and equivalent current density. And the expression can be expressed as,

15 $$ B = B\left( {{\varvec{r}},R_{1} ,R_{2} ,\varphi ,h,J} \right) $$

where, *R*_*1*_ reflects the radial position of the permanent magnet; *R*_*2*_*-R*_*1*_ can be expressed as the radial length of the permanent magnet. The angle φ of the permanent magnet can be converted into a magnetic pole number.

### Magnetic induction intensity expression

In a permanent magnet governor, n permanent magnets of the same size and opposite polarity are arranged in a circle (*n* is an even number, *n* = *2k*). The magnetic induction of the n permanent magnets at point P can be obtained by the following coordinate transformation.

16 $$ \left\{ {\begin{array}{*{20}c} {\rho_{0} = \rho_{0} } \\ {\theta_{0} = \theta_{0} + \left( {m - 1} \right)\alpha } \\ {z_{0} = z_{0} } \\ \end{array} } \right. $$

where, m represents the m-th permanent magnet and α is the angle between the two permanent magnets.

After that, the magnetic field distribution in the external space of each permanent magnet is obtained. The analytical equation of the magnetic field generated by n permanent magnets at any point in their external space is obtained by superposition. If two adjacent permanent magnets have opposite polarity, then the three components of the magnetic field are expressed as follows.

17 $$ \left\{ \begin{gathered} {\varvec{B}}_{{\varvec{x}}} = \mathop \sum \limits_{m = 1}^{k} B_{x} \left( {2m - 1} \right) - \mathop \sum \limits_{m = 1}^{k} B_{x} \left( {2m} \right) \hfill \\ {\varvec{B}}_{{\varvec{y}}} = \mathop \sum \limits_{m = 1}^{k} B_{y} \left( {2m - 1} \right) - \mathop \sum \limits_{m = 1}^{k} B_{y} \left( {2m} \right) \hfill \\ {\varvec{B}}_{{\varvec{z}}} = \mathop \sum \limits_{m = 1}^{k} B_{z} \left( {2m - 1} \right) - \mathop \sum \limits_{m = 1}^{k} B_{z} \left( {2m} \right) \hfill \\ \end{gathered} \right. $$

where, *B(m)* is the magnetic induction intensity generated by the m-th permanent magnet.

## Simulation analysis and results discussion

Previous experimental studies have shown that the numerical solution of the finite element method is the more accurate method when calculating the magnetic induction intensity of permanent magnets^13,14^. To verify the correctness of the analytical formula derived by the molecular current method, and to analyze the effect of each parameter of the permanent magnet on the magnetic induction intensity, the analytical solution of the calculation is compared with the numerical solution of the Maxwell finite element method through an arithmetic analysis. The parameters of the sector permanent magnets are shown in Table 1.

**Table 1:** Permanent magnet parameters.

Performance or dimensional parameters	Numerical value
Material	N35
Remanence* B*_r_(T)	1.23
Coercivity* H*_c_(KA/m)	890
Inner diameter *R*_1_(mm)	30
Outer diameter *R*_2_(mm)	40
Height *h*(mm)	10
Angle *φ*(rad)	$\pi /3$

**Figure 4:**
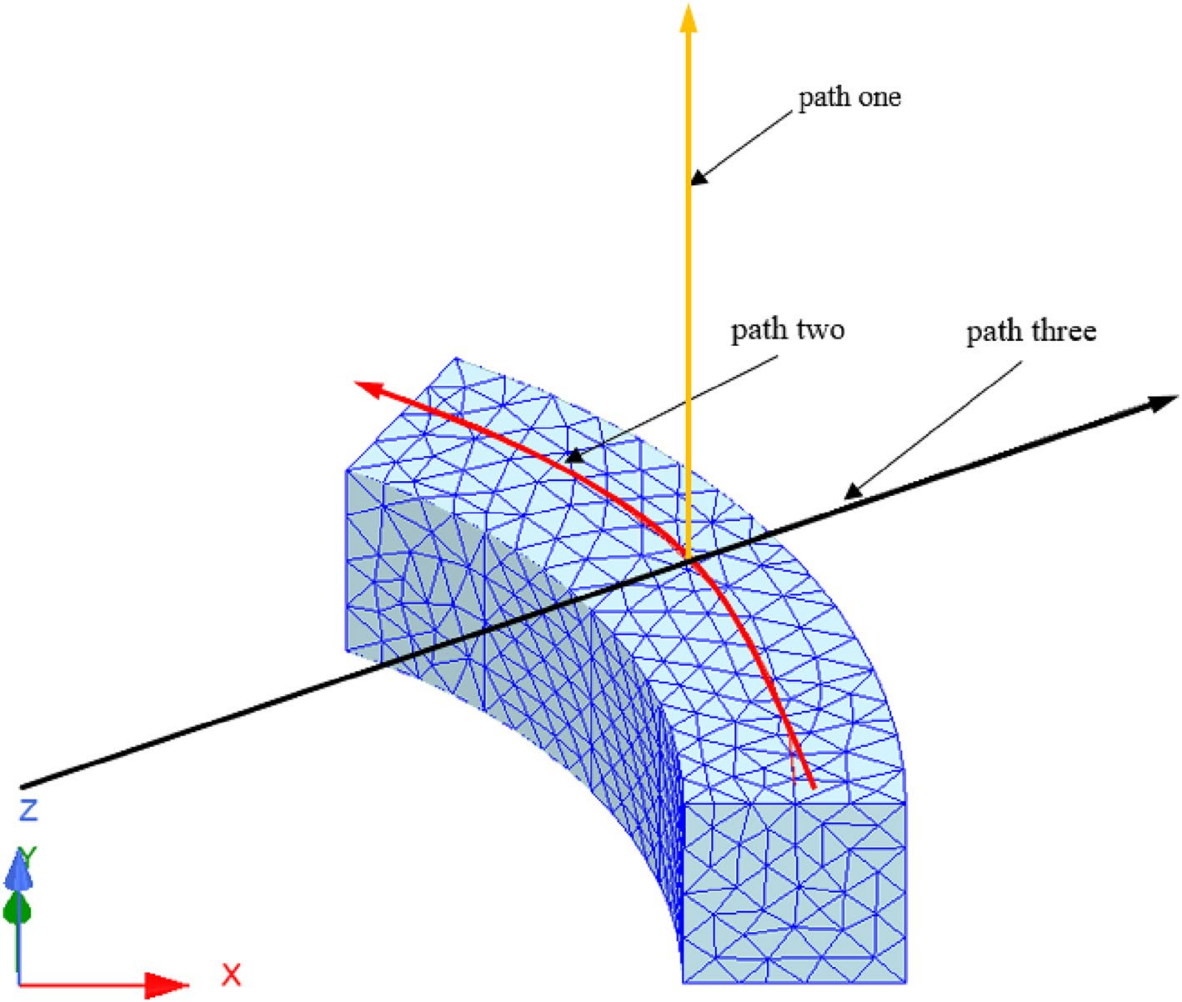
Spatial coordinate system of sector permanent magnet.

**Figure 5:**
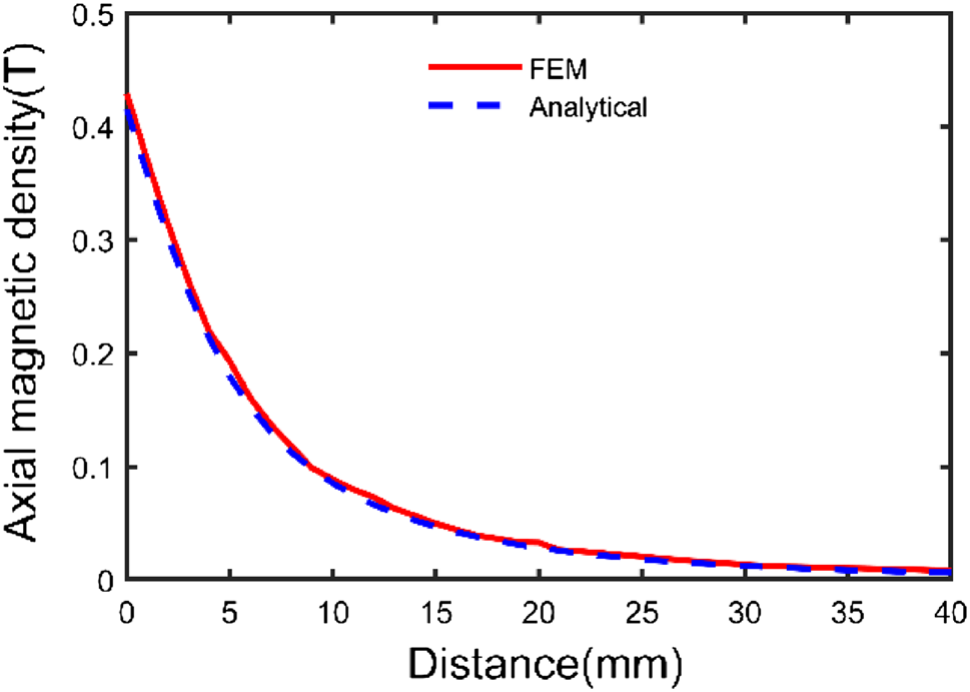
Axial component variation curve of path one.

**Figure 6:**
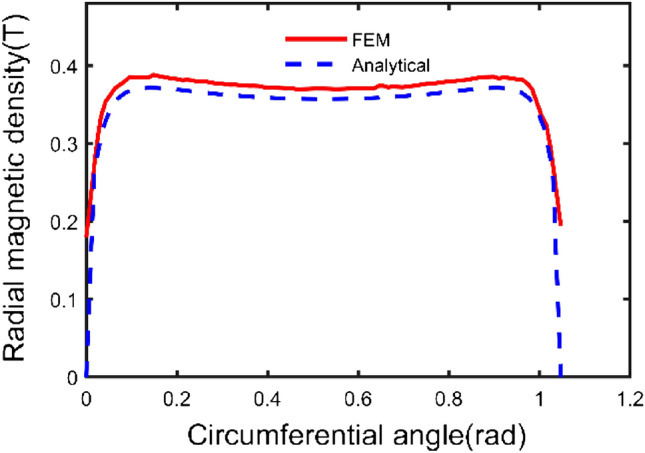
Axial component variation curve of path two.

**Figure 7:**
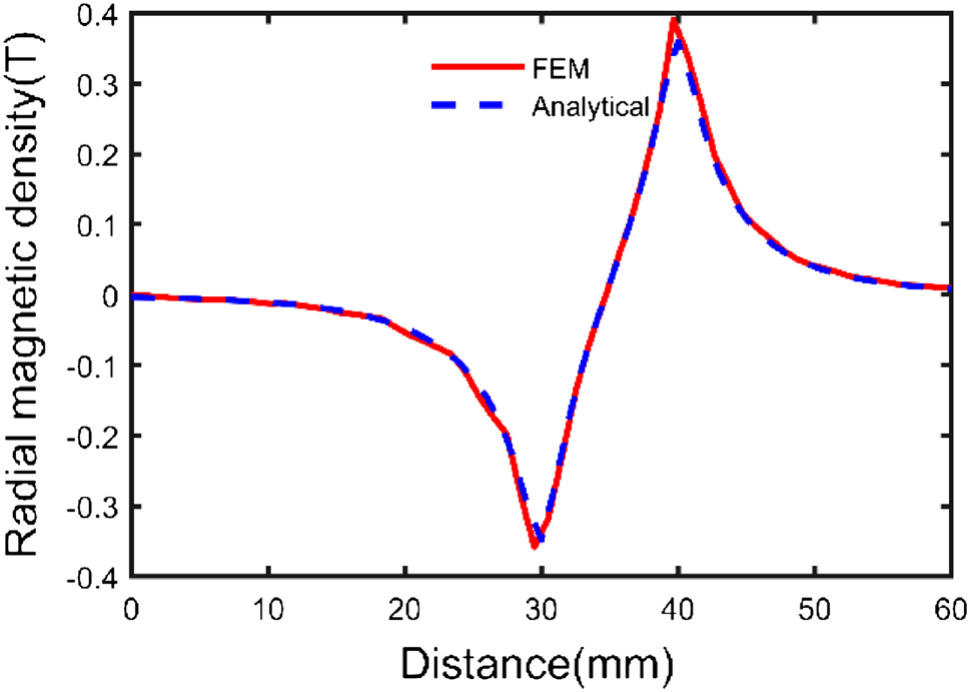
Radial component variation curve of path three.

According to the parameters in Table 1, a three-dimensional model is shown in Fig. 4. Based on the above theoretical derivation, the axial components of path one and path two and the radial component of path three have been calculated by the integral2 function in MATLAB, respectively. The results are shown in Figs. 5, 6, and 7.

From Figs. 5 and 6 with the increase of the distance from the surface of the permanent magnet, the trend of the axial component of the magnetic induction intensity is as follows: firstly, the axial component of the magnetic induction intensity is maximum at the end face of the permanent magnet. It shows a decaying trend with the increase of the distance from the surface of the permanent magnet. It indicates that the air gap size is the main reason for the mechanical characteristics of the Permanent magnets^15^. Secondly, the distribution of the axial component of the air-gap magnetic induction intensity in the same plane transversely has a special relationship with the distance. At a distance less than a specific value, the value of magnetic induction intensity at the central region is lower than that of the edge region.

From Fig. 7, the radial direction, the radial component of the air gap flux density decreases gradually as the distance from the inner and outer sides of the permanent magnet increases.

As seen from the figures, the calculation results of the two methods are consistent, and the maximum deviation point is at the end of the permanent magnet, which is only 6.06%. The analysis method is accurate and effective.

## Optimized design of permanent magnet parameters

This paper takes the above permanent magnet parameters as an example in the permanent magnet governor, and uses the analytical formula and Maxwell software to study the influence of the permanent magnet structure parameters on its air gap magnetic induction strength, and analyzes the selection principle of the main parameters of the permanent magnet of the disk permanent magnet governor, and summarizes its regular conclusions, and provides a basis for the optimal design of the permanent magnet governor. Since the axial component of the magnetic field has a direct impact on the transmission performance, the radial component is minor, and the distribution changes are more complicated, so the calculation is carried out with the axial component.

### Parameter influence

To comprehensively investigate the relationship between parameter and axial magnetic density, the structural parameters are radial position, radial length, thickness, and number of magnetic pole pairs. The middle radius of the permanent magnet and the air gap of 1 mm is chosen as the reference point. To study the variation law of each structural parameter on the axial component of the magnetic induction intensity and to provide a basis for structural optimization.

**Figure 8:**
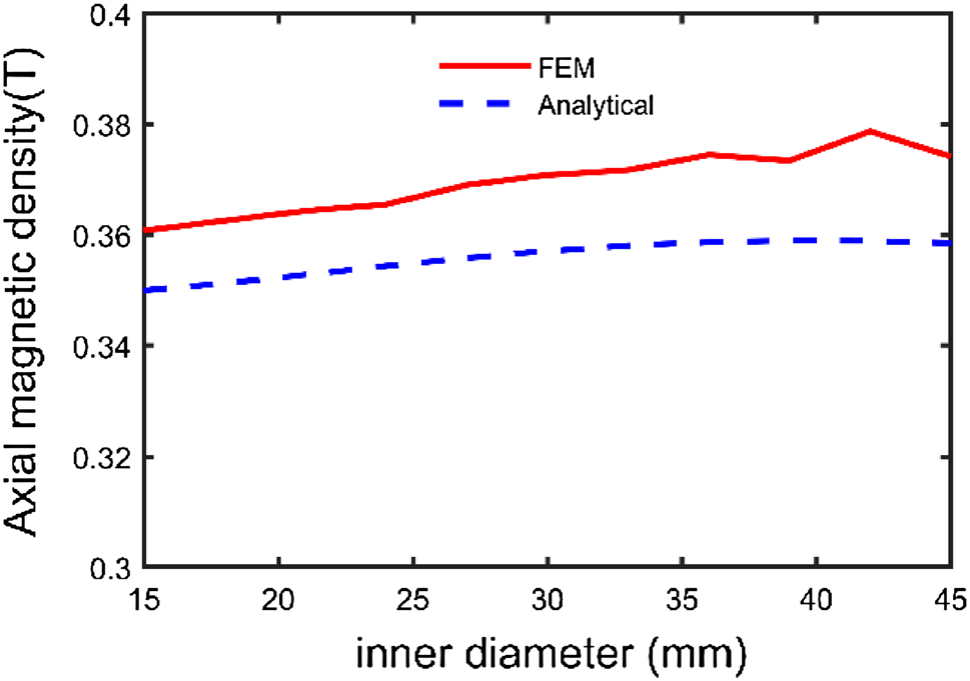
Effect of radial position on the axial component of the air gap flux density.

From Fig. 8, when the cross-sectional area and thickness of the permanent magnet remain unchanged, the magnetic induction intensity of the air gap does not change significantly when the radial position of the permanent magnet is changed. The reason is that changes in the shape and position of the section do not affect the magnetic potential. Therefore, the permanent magnets with different cross-sectional shapes are equal. The principle is that the parameters, such as the cross-sectional area and thickness of the permanent magnets, remain unchanged before and after equating. The transmission performance of the governor has little influence^16,17^.

**Figure 9:**
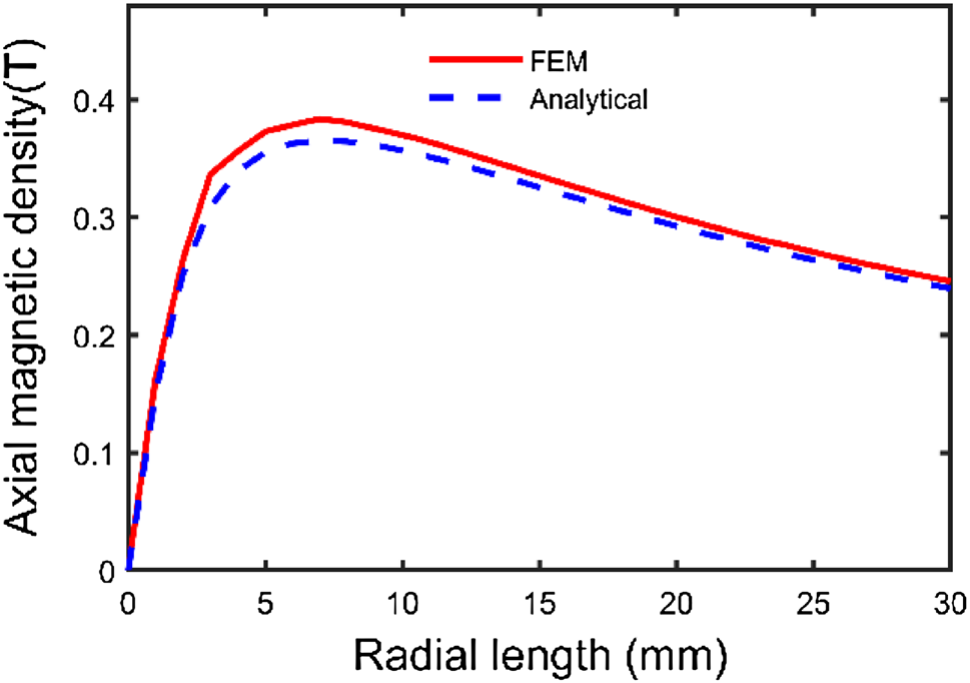
Effect of radial length on the axial component of the air gap flux density.

From Fig. 9, when the radial length of the permanent magnet is around 9 mm, the air gap flux density reaches its maximum. As the radial length of the permanent magnet increases, the air gap flux density gradually decreases. The radial length of the permanent magnet mainly affects the radial component of the air-gap magnetic inductance, and the influence on the tangential and axial components is relatively small. Therefore, the air gap magnetic induction can be increased by reducing the radial length.

**Figure 10:**
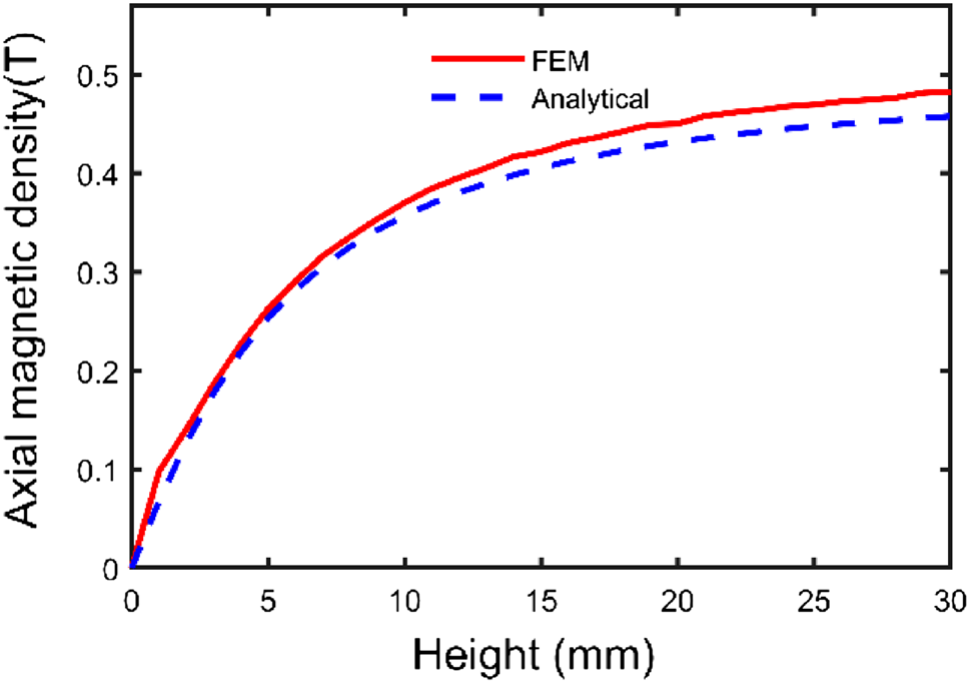
Effect of thickness on the axial component of the air gap flux density.

From Fig. 10, with the increase of permanent magnet thickness, the air gap flux density gradually increases, and the gradient of the rising curve gradually decreases. Finally, the air gap flux density tends to be stable. The reason is that the magnetoresistance and magneto dynamic potential of the permanent magnet simultaneously increases, which decreases the air gap flux, and the increase rate of the air gap flux density decreases. Therefore, the thickness needs to be selected reasonably to utilize the magnetic energy effectively.

**Figure 11:**
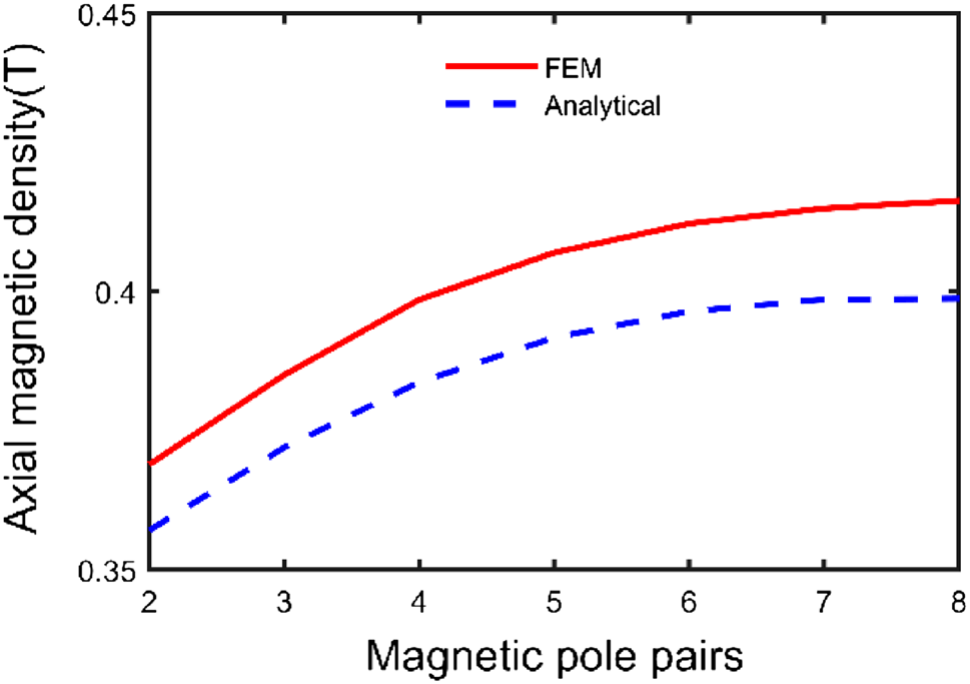
Effect of pole pairs number on the axial component of the air gap flux density.

The influence law of the number of magnetic pole pairs on the magnetic induction intensity is shown in Fig. 11. When the number of pole pairs is eight, the air gap flux density is maximum. Because when the duty cycle is constant, the number of pole pairs increases, the angle of each permanent magnet decreases, and the magnetic induction lines become more concentrated, which enhances the air gap flux density.

### Results and discussion

Considering the permanent magnet number and the air gap flux density, the optimized structure parameters of permanent magnet governor are: inner diameter $R_{1}$ = 26 mm, outer diameter $R_{2}$ = 35 mm, height *h* = 12 mm, angle φ = $\pi /12$. The optimized structure is analyzed and calculated, and the results before and after optimization are compared (the permanent magnet performance parameters remain unchanged), the comparison results are shown in Table 2. The amount of permanent magnet before the optimization is 14,667 mm^3^, and the air gap flux density is 0.357 T; the amount of permanent magnet after optimization is 13,798 mm^3^, and the air gap flux density is 0.408 T. The amount of permanent magnet after optimization is reduced by 7.84%, and the axial component of air gap flux density is increased by 14.3%.

**Table 2:** Comparison of parameters before and after optimization of permanent magnets.

Dimension parameter	Before optimization	After optimization
Inner diameter $R_{1}$ (mm)	30	26
Outer diameter $R_{2}$ (mm)	40	35
Height $h$ (mm)	10	12
Angle $\varphi$ (rad)	$\pi /3$	$\pi /12$
Volume (mm^3^)	14,667	13,798
Axial component of the air gap flux density (T)	0.357	0.408

### Experimental verification

**Figure 12:**
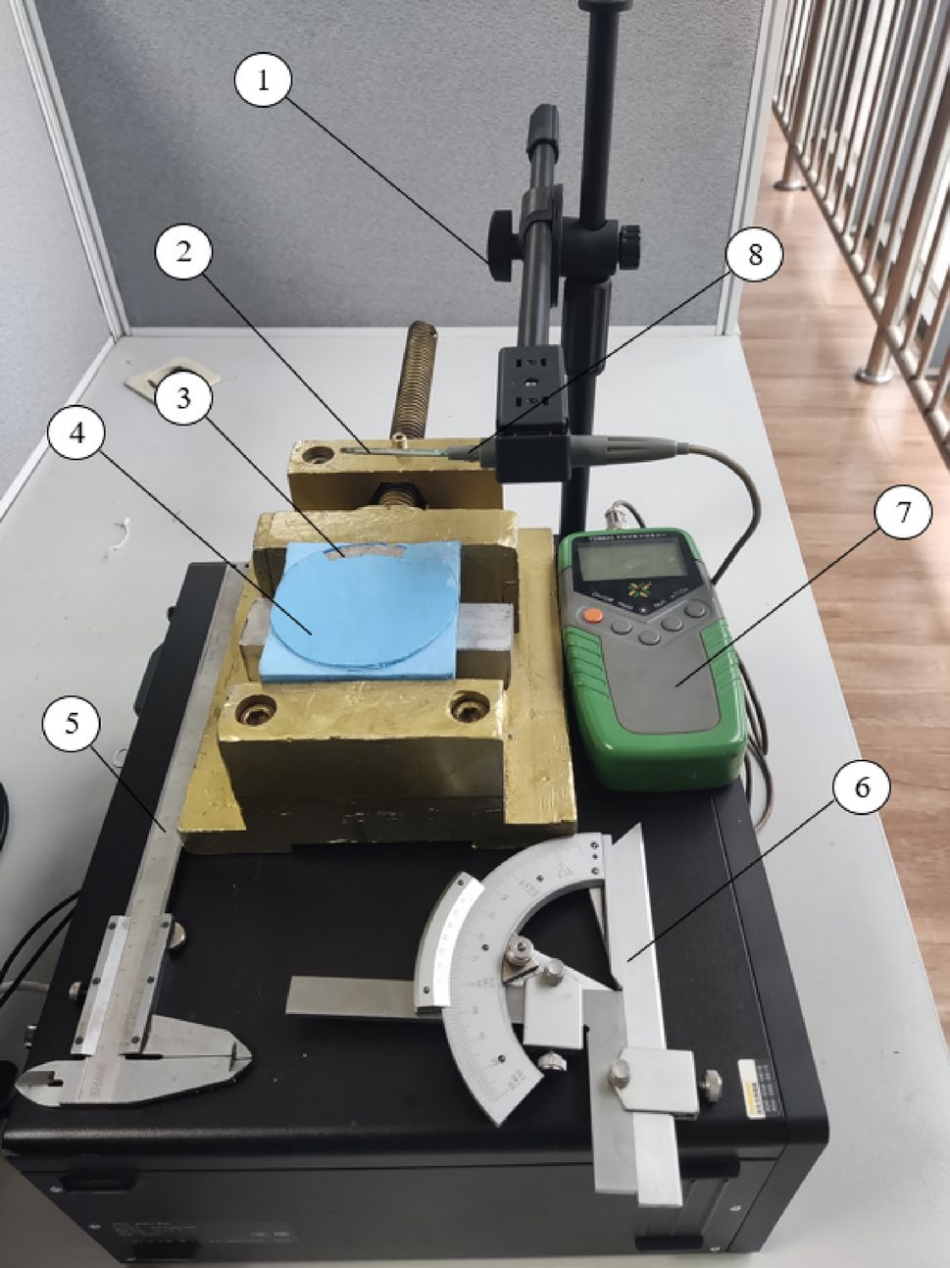
Measurement test of magnetic induction strength of permanent magnets. ①lifting platform ② aluminum bench vice ③ permanent magnet ④ permanent magnet holder ⑤ vernier caliper ⑥ cursor universal angle ruler ⑦ handheld Tesla meter ⑧ Tesla meter probe.

The sector permanent magnets are mounted for measurement tests. Figure 12 shows the test device. To measure the magnetic induction intensity of two sizes of permanent magnets in different spatial positions before and after optimization, the position of the measurement point was changed by adjusting the height of the elevating table. The magnetic induction intensity of permanent magnets was measured several times in different spatial positions, and the average value was recorded. The permanent magnet is mounted on the permanent magnet holder and clamped by an aluminum bench vice, and then the Tesla meter probe is fixed on the lifting table. In the test, a cursor universal angle ruler and a vernier caliper are used to ensure that the Tesla meter probe is aligned with the center of the permanent magnet, and a vernier caliper measures the distance between the Tesla meter probe and the upper surface of the permanent magnet. The magnitude of the magnetic induction intensity at the current position can be read from the handheld Tesla meter. By moving the platform height, the magnetic induction intensity of the sector permanent magnets is obtained in different spatial positions before and after optimization.

**Figure 13:**
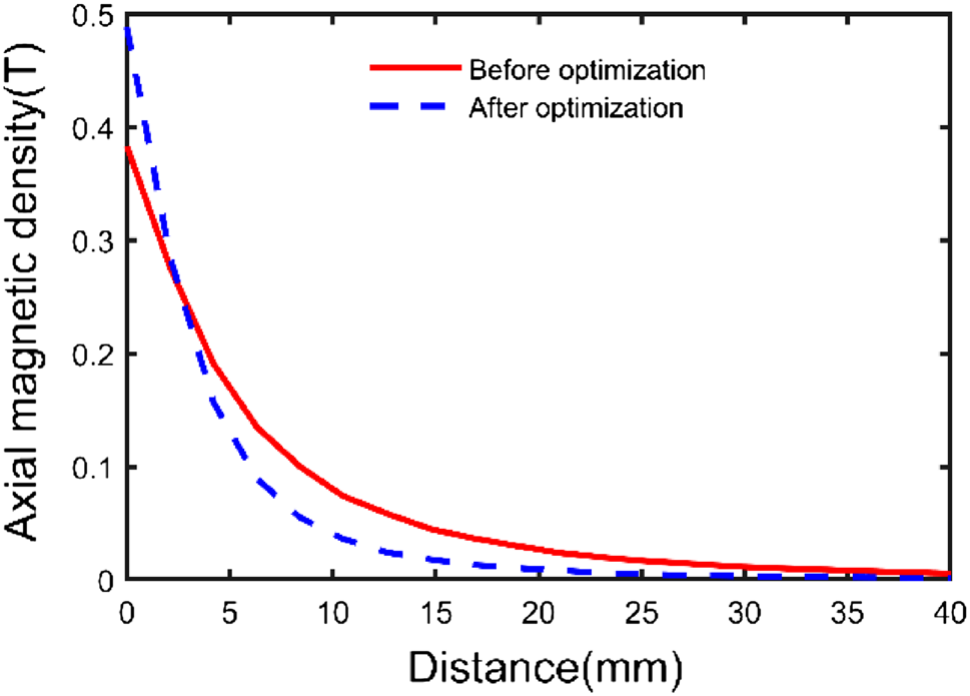
Experimental results comparison before and after optimization.

Figure 13 shows the variation curve of magnetic induction intensity measured by the sector permanent magnet at different heights obtained from the above experimental process.

The test results show that the magnetic induction intensity decreases with the in-crease in distance, and there is an intersection point at a distance of 2.5 mm. When the distance from the upper surface is less than 2.5 mm, the magnetic induction intensity after optimization is more significant than that before optimization. When the distance from the upper surface is greater than 2.5 mm, the magnetic induction intensity is greater than that after optimization. The optimization effect is most obvious on the upper surface of the permanent magnet, and the magnetic induction intensity after optimization is increased by 27.5% compared with that before optimization. Therefore, the optimized parameters using the analytical formula by the molecular current method can effectively improve the magnetic induction strength of the permanent magnet, which can guide the preliminary design and later product improvement.

## Conclusion

This paper calculates the spatial magnetic field distribution of the sector permanent magnet in the permanent magnet governor based on the molecular current method. The correctness of the solution is verified by the numerical analysis and comparison with the finite element method. According to the analytic formula, the following conclusions can be drawn:

1. Without considering the number of permanent magnets and limiting the outer diameter of the disk-type permanent magnet governor, the magnetic induction intensity of the air gap does not change significantly with the radial position of permanent magnets. The radial length of the permanent magnet mainly affects the radial component of the magnetic induction, while the effect on the tangential and axial components is relatively small. The magnetic induction intensity at the air gap increases with the thickness in-creasing of permanent magnets, and stabilizes after increasing to a specific value. The magnetic density of the air gap increases with the increase in the number of pole pairs.
2. When considering the number of permanent magnets, it is required not to exceed the number of permanent magnets in the initial structure. After the structure optimization, the axial magnetic field strength is increased by 14.3% compared with the original one. It provides a theoretical reference for optimizing sector permanent magnets in permanent magnet governor.

## Data Availability

The datasets analyzed during the current study available from the corresponding author on reasonable request.
